# Surgical management of compound odontoma associated with unerupted tooth: a case report

**DOI:** 10.11604/pamj.2022.43.108.34898

**Published:** 2022-10-28

**Authors:** Fatima Ezzahra Zidane, Youssra Azzouz, Rachid Fawzi

**Affiliations:** 1 International University of Rabat, College of Health Sciences, International Faculty of Dental Medicine, Rabat, Morocco,; 2 Private University Marrakech- Marrakesh University Private Hospital-Dental Department, Marrakesh, Morocco

**Keywords:** Odontoma, compound, unerupted, tumor-like, case report

## Abstract

Compound odontoma has been reported to be the most common of all odontogenic neoplasms and tumor- like lesions. It is a slow-growing, asymptomatic neoplasms found incidentally during a routine radiography examination. In general, the clinical indicators of odontoma may include eruption disturbance (non-eruption of permanent teeth, retention of deciduous teeth), expansion of the cortical bone, teeth malposition and pain. In this case, the presence of odontoma prevented the physiological eruption of permanent mandible incisor. We describe the surgical procedure to remove a compound odontoma of 21 small tooth-like structures localized in the mandible of a child boy associated with an unerupted permanent mandible incisor.

## Introduction

Odontomas are the most common odontogenic tumors, representing 70% of all odontogenic tumors [1]. The term odontoma was coined by Paul Broca in 1867 [2]. They are considered to be hamartomas rather than neoplasms and are composed of the tissues native of teeth: enamel, dentin, cementum and pulp tissue [3]. The 4^th^ edition of the World Health Organization´s Classification (WHO classification) of odontogenic tumors published in January of 2017 divides these tumors into complex and compound odontoma. It should be noted that a mixed form can also be found associating compound structures within complex structures. During the development of the tumor, if the enamel and dentin are deposited in such a pattern that the resulting structure is anatomically similar to normal teeth, in such cases, the lesion is classified as a compound odontoma. They can occur at any age but are most common in the first two decades of life. There is no gender predilection and more common in the maxilla, especially the anterior maxilla, than in the mandible [4,5]. The aim of this case report was to describe the surgical procedure to remove a compound odontoma localized in the mandible of a child boy associated with an unerupted permanent mandible incisor.

## Patient and observation

**Patient information:** a 12-year-old boy in apparent good health presented to the pediatric department at International Clinic of Dentistry of International University of Rabat, for absence of eruption of 31 as reason of consultation.

**Figure 1:**
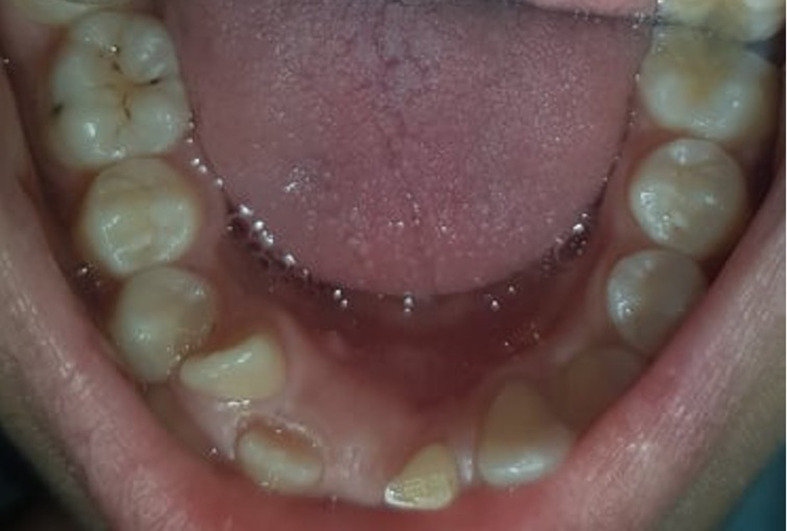
bump on the lingual surface in the mandibular region, lingo version and rotation of 80 degrees of the lower right lateral incisor 42, persistence of the left lower central incisor 71

**Clinical findings:** intraoral examination revealed no gingival swelling and no symptoms in the affected area. But a bump on the lingual surface in the mandibular region, a lingo version and rotation of 80 degrees of the lower right lateral incisor 42 and persistence of the left lower central incisor 71, were observed (Figure 1).

**Figure 2:**
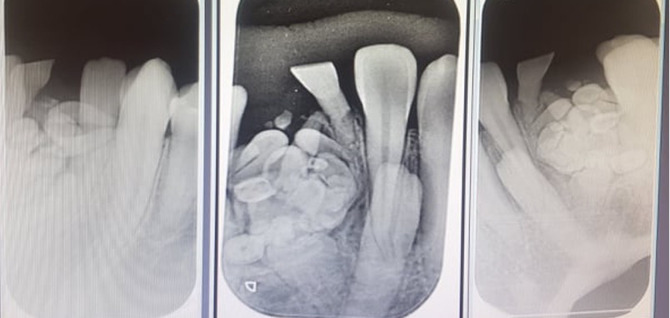
periapical radiography showing mixed radio-opaque and radiolucent lesion in the region of 71, 42 surrounded by a thin radiolucent band

**Diagnostic assessment:** periapical radiography findings showed mixed radio-opaque and radiolucent lesion in the region of 71, 42 surrounded by a thin radiolucent band (Figure 2).

**Diagnosis:** considering the clinical and radiographic presentation, a radiographic diagnosis of compound odontoma was determined.

**Figure 3:**
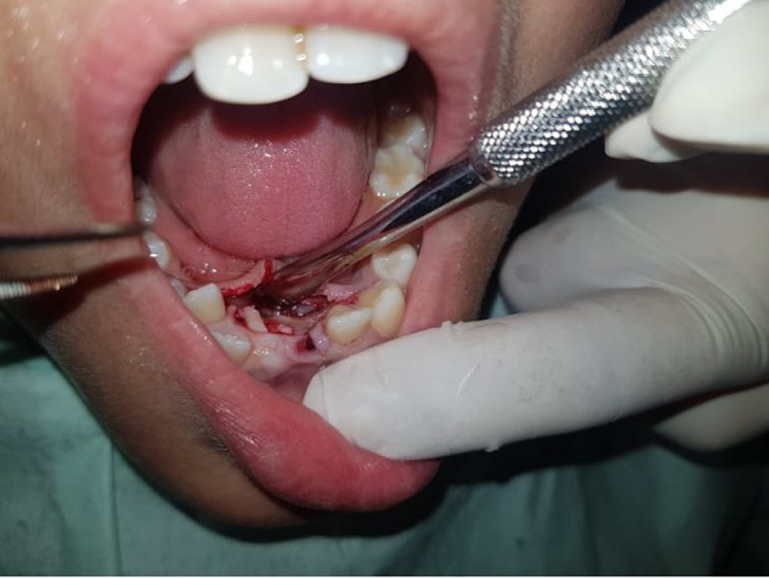
bone eviction to materialize odontomas

**Figure 4:**
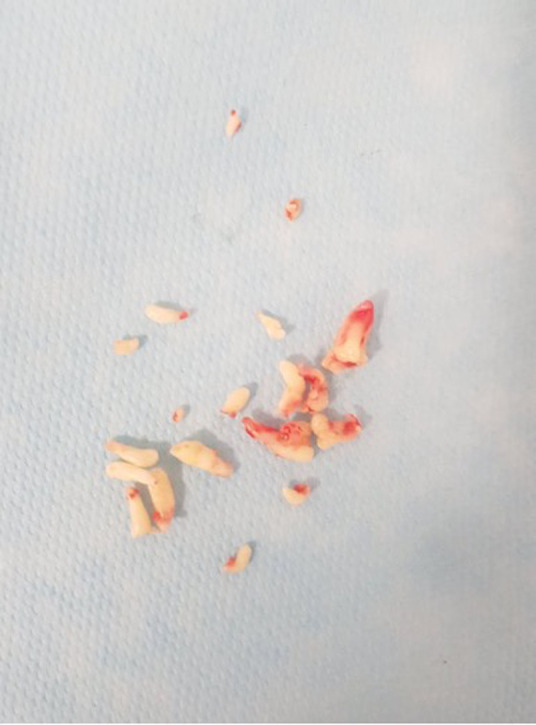
twenty-one (21) small tooth-like structures

**Figure 5:**
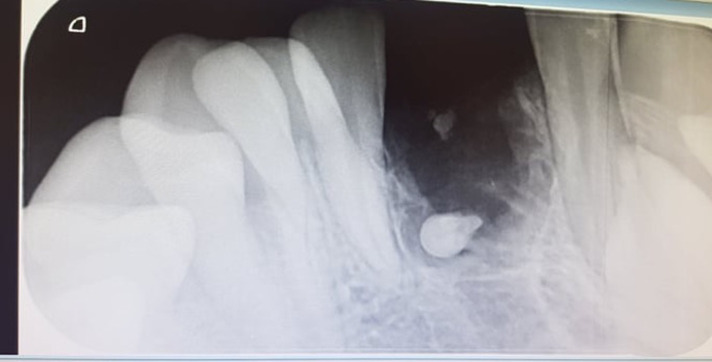
X-ray after 2 years

**Therapeutic interventions:** under local anesthesia, we have carried out the extraction of the 71, after that we started the eviction surgery of the odontoma, we tried to undertook a minimally invasive surgical removal, for that we started first with an incision and a detachment using a surgical stripper, after that an osteotomy using a bone burr, finally, after bone eviction we were able to materialize our odontomas and had started the harvest (Figure 3). The resected tumor was found to consist of 21 small tooth-like structures (Figure 4). Before sutures, we did an X-ray control and found one last odontoma at the bottom, but since the patient was uncooperative, we could not extract it (Figure 5). Sutures were made, a clinical control after two weeks has been established.

**Figure 6:**
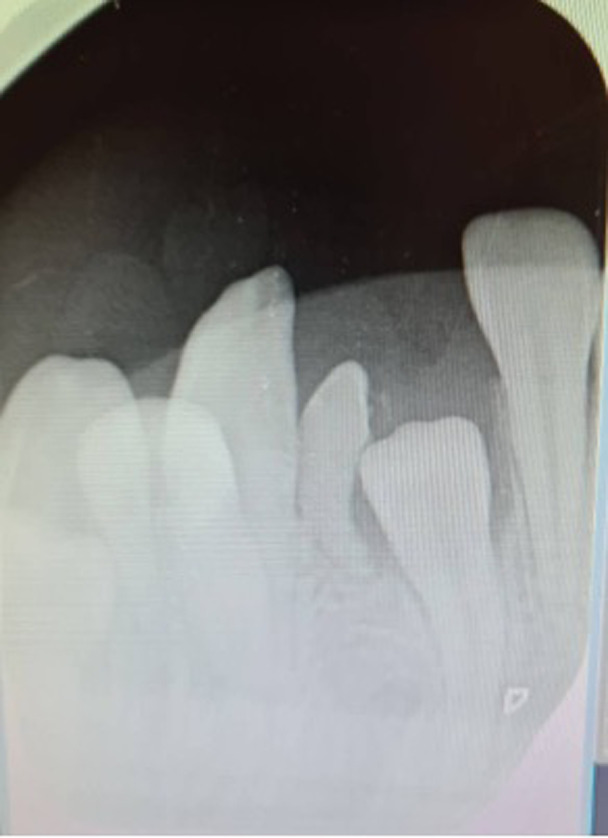
X-ray control, one last odontoma at the bottom

**Figure 7:**
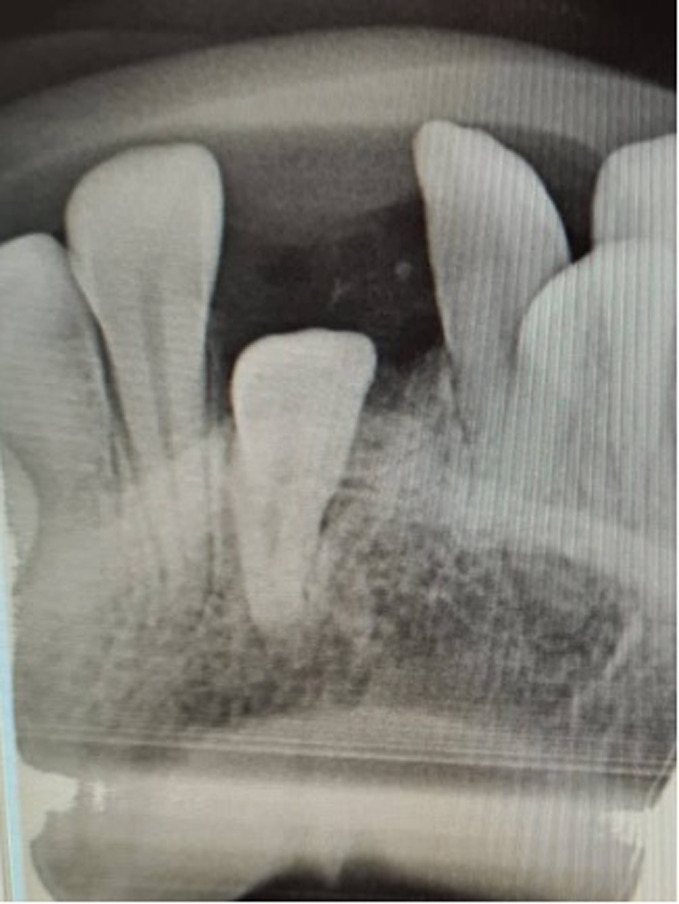
extraction of the final odontoma and 31 was about to erupt

**Follow-up and outcome of interventions:** two years after removal of the odontoma, we received the patient, we noticed during our clinical examination, a small crown on the eruption site of the 31. We took an X-ray, on our radiograph it turned out that it was the last odontoma that we had not been able to extract, but it evolved and grew in size (Figure 6). The decision to extract the odontoma was taken, moreover we were able to materialize the presence of the 31 in the process of eruption and since it presented the last obstacle before the eruption of the 31, we had performed the extraction of the last odontoma (Figure 7).

**Patient perspective:** though dentition improvement was expected and the 31 was about to erupt (Figure 7). We aim to start an orthodontic treatment.

**Informed consent:** after talking to the mother of the child, we were able to convince her to let us publish the case of her son.

## Discussion

Compound odontoma has been reported to be the most common of all odontogenic neoplasms and tumor- like lesions. It is a slow-growing, asymptomatic neoplasms found incidentally during a routine radiography examination [6]. They are generally small; however, they may occasionally grow large, resulting in bone expansion. Compound odontoma is usually located in the anterior region of the maxilla, over the crown of erupting tooth or between the roots of erupted teeth. In about 80% of cases, they are associated with impacted or unerupted teeth [7]. In general, the clinical indicators of odontoma may include eruption disturbance (noneruption of permanent teeth, retention of deciduous teeth), expansion of the cortical bone, teeth malposition and pain. In this case, the presence of odontoma prevented the physiological eruption of permanent mandible incisor.

The etiology of odontoma remains unknown. Several theories have been proposed, and various causes including trauma to the primary dentition, infection, family history and genetic mutation, odontoblastic hyperactivity have been postulated [8,9]. Some hereditary anomalies can also show odontomas such as Gardner's syndrome and Hermann's syndrome [9]. Usually, the compound odontoma appears as a collection of miniature tooth-like structure surrounded by a narrow radiolucent zone 2, 9. It can be confused with any other entity such as supernumerary teeth. Histologically, the compound odontoma is composed of dentin, cementum, pulpal tissue and enamel arranged in an organized manner of dental structures and partially surrounded by a connective tissue capsule [10]. The treatment of choice has been conservative surgical removal, depending on the size and location of the odontoma, followed by histopathological analysis to confirm the diagnosis. After excision, bone grafts may be necessary depending on the need for further treatment, or the size and location of the odontoma. The odontoma once enucleated the recurrence is very low but literature suggested in young children close monitoring is necessary. A careful follow-up of the case, implementing preventive and interceptive orthodontics, if necessary, prevents future malocclusion [11-13].

## Conclusion

In conclusion, the presence of odontoma in association with the impacted teeth needs an early diagnosis and a surgical removal treatment to prevent eruption disturbances.
